# Supplementary material for: Gradual Loss of Social Group Support during Competition Activates Anterior TPJ and Insula but Deactivates Default Mode Network
Source: Brain Sci. 2023 Oct 25;13(11):1509. doi: 10.3390/brainsci13111509 (PMC10669722; doi:10.3390/brainsci13111509)
Supplement: Supplementary file 1 [file brainsci-13-01509-s001.zip › brainsci-2585971-supplementary.pdf]

Sociodemographic and Psychometric Characteristics

Supplementary Table S1. Sociodemographic and Psychometric Characteristics of the Subjects

Variables			High-support phase	Ostracism phase	Comparison
	n (%)	Mean ± SD (min-max)	Mean ± SD (min-max)	Mean ± SD (min-max)	
Age	N/A	21.7±1.6 (18-24)	N/A	N/A	N/A
Gender<br>Female<br>Male	 10 (55.6)<br>8 (44.4)	N/A	N/A	N/A	N/A
Rosenberg Self-Esteem Scale	N/A	21.6 ± 3.6 (16-27)	N/A	N/A	N/A
Multidimensional Scale of Perceived Social Support	N/A	64.6±13.8 (43-84)	N/A	N/A	N/A
Inventory of Socially Supportive Behaviors	N/A	141.8±25.9 (95-194)	N/A	N/A	N/A
Need-Threat Scale	Subscale				
	Belonging		3.75±.74 (2.80-5.00)	2.91±1.00 (1.00-4.60)	Z= -3.082 p=.002
	Self-Esteem		3.92±.71 (2.80-5.00)	2.60±.79 (1.00-4.00)	Z = -3.415 p=.001
	Meaning of existence		4.06±.70 (2.60-5.00)	3.13±.88 (1.00-4.60)	Z = -2.923 p=.003
	Control		3.04±.75 (1.00-4.20)	2.55±.76 (1.20-3.60)	Z = -2.885 p=.004
Emotional status[a]			*Before the Study*	*After the Study*	
	Motivation		8.58±.1.46 (6-10)	8.29± 1.79 (5-10)	Z= -.406 p=.685
	Happiness		8.47±1.84 (5-10)	8.70±1.79 (4-10)	Z = -.731 p=.465
	Sadness		1.58± 1.06 (1-5)	2.23± 1.88 (1-7)	Z = -1.420 p=.156
	Fear		3.00±2.44 (1-8)	2.11±2.44 (1-10)	Z = -1.231 p=.218
	Anger		1.11±.33 (1-2)	1.52 ± 1.28 (1-6)	Z = -1.289 p=.197

[a]VAS: Visual analog scale (1-10), N/A: Not applicable

Supplementary Table S2. Distribution of the Images Used in the Experiment According to the Difficulty Level

Image Group	Correct or Incorrect Answers		Score Difference	The number and percentage of images used in the HSP	The number and percentage of images used in the FP	The number and percentage of images used in the OP
1	✓	✓	<1	5 20%	5 20%	5 20%
2	✓	×	<1	4 16%	5 20%	6 24%
3	×	✓	<1	7 28%	3 12%	5 20%
4	×	×	<1	5 20 %	5 20%	5 20%
5	✓	✓	>3	1 4%	2 8%	1 4%
6	✓	×	>3	0	1 4%	2 8%
7	×	✓	>3	2 8%	2 8%	0
8	×	×	>3	1 4%	2 8%	1 4%
				Total difficult image ratio: 84%<br>easy image ratio: 16%<br>Win rate in difficult images: 72% lose: 12%	Total difficult image ratio: 72%<br>easy image ratio: 28%<br>Win rate in difficult images: 40% lose: 32%	Total difficult image ratio: 84%<br>easy image ratio: 16%<br>Win rate in difficult images: 12% lose: 72%
